# Extramedullary plasmacytoma of the nasopharynx: A case report and review of the literature

**DOI:** 10.3892/ol.2013.1712

**Published:** 2013-11-27

**Authors:** YEN-LIANG CHANG, PO-YUEH CHEN, SHIH-HAN HUNG

**Affiliations:** 1 Department of Otolaryngology Head & Neck Surgery, Cathay General Hospital, Taipei, Taiwan, R.O.C.; 2 School of Medicine, Fu Jen Catholic University, Taipei, Taiwan, R.O.C.; 3 Department of Otolaryngology Head & Neck Surgery, Taipei Medical University-Shuang Ho Hospital, Taipei, Taiwan, R.O.C.; 4 Department of Otolaryngology, Taipei Medical University Hospital, Taipei, Taiwan, R.O.C.; 5 Department of Otolaryngology, School of Medicine, Taipei Medical University, Taipei, Taiwan, R.O.C.

**Keywords:** plasmacytoma, extramedullary, head and neck, nasopharynx

## Abstract

Plasmacytoma is an extremely rare and discrete solitary mass of neoplastic monoclonal plasma cells. Extramedullary plasmacytoma (EMP) tends to occur during the fifth and seventh decades of life and is rarely diagnosed in younger patients. Only four cases of EMP have been previously reported in relatively young patients. Here we report a 15-year-old patient presenting with long-term nasal obstruction, who was found to have EMP of the nasopharynx. The patient was treated with surgery followed by radiotherapy with a satisfactory outcome. To the best of our knowledge, this study describes the the youngest individual with nasopharyngeal EMP to be reported in the literature.

## Introduction

Plasmacytoma is an extremely rare and discrete solitary mass of neoplastic monoclonal plasma cells, which was first described by Schridde in 1905 (1). Extramedullary plasmacytoma (EMP) has been seldom reported and accounts for 4% of all non-epithelial tumors of the upper respiratory tract (2). While occasionally localized to the gastrointestinal tract, lungs, mammae, testes and skin, it has been previously reported that 80% of EMPs are localized in the head and neck region (3,4). Common clinical symptoms include epistaxis, rhinorrhea, a sore throat, dysphonia and hemoptysis (5,6). EMP is usually managed through radiotherapy, with or without surgery. The current study presents the case of a young male patient with EMP of the nasopharynx who was treated successfully with surgery and radiotherapy. Written informed consent was obtained from the patient.

## Case report

**Figure 1:**
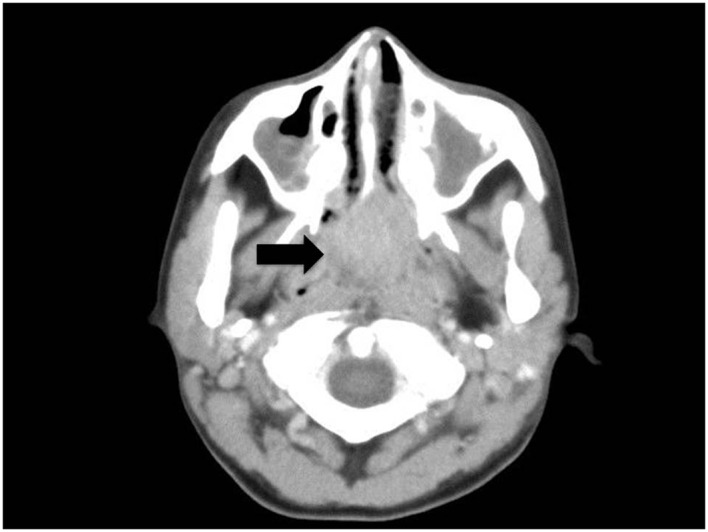
CT revealing a mass (arrow) occupying almost the entire nasopharyngeal space. CT, computed tomography.

**Figure 2:**
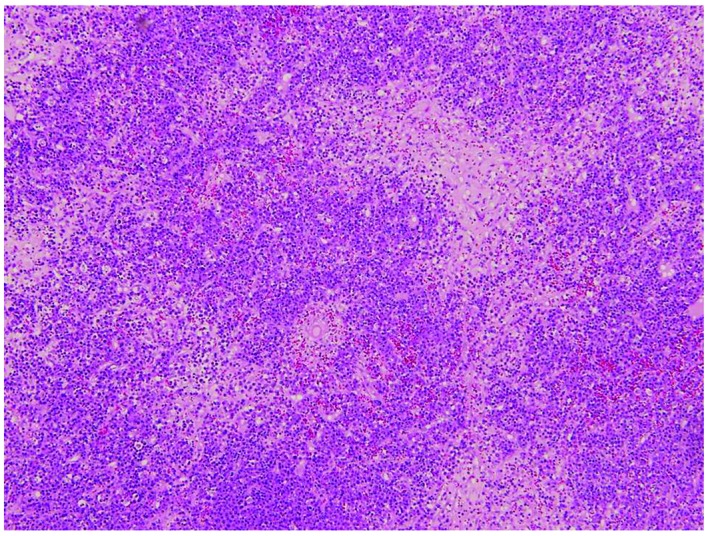
Microscopically, the tumor showed sheets of monomorphic round-to-oval cells with nuclear pleomorphism (hematoxylin and eosin; magnification, ×100).

**Figure 3:**
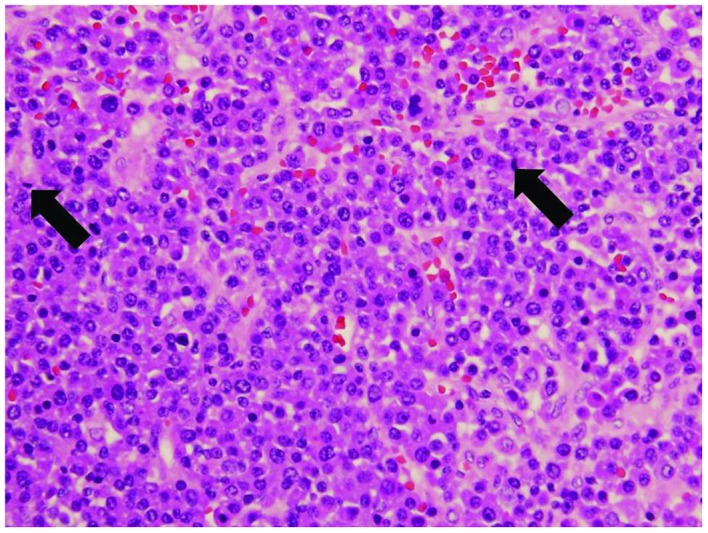
Microscopically, the tumor showed sheets of monomorphic round-to-oval cells (arrows) with nuclear pleomorphism (hematoxylin and eosin; magnification, ×400).

**Figure 4:**
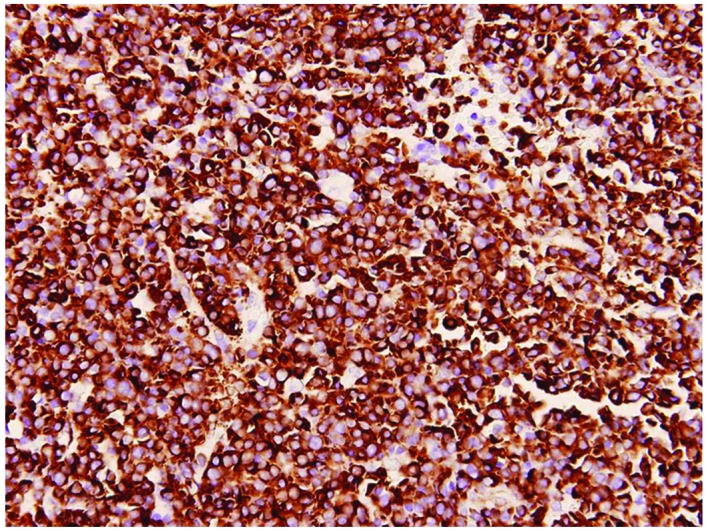
Tumor cells positive for VS38c (magnification, ×400).

A 15-year-old male was referred to the Department of Otolaryngology, Cathay General Hospital (Taipei, Taiwan) due to intermittent epistaxis lasting for 2 weeks. In addition, the patient reported a 3-year history of persistent nasal obstruction. A physical examination revealed an extremely large tumor in the center of the nasopharynx that bled easily when touched. Computed tomography revealed a mass occupying almost the entire nasopharyngeal space without involvement of the bony structures (Fig. 1). Surgical treatment was arranged and the tumor was excised completely using a transpalatal approach. Microscopically, the tumor showed sheets of monomorphic round-to-oval cells with eccentric nuclei and a dense infiltration of plasmacytoid cells (Fig. 2). Significant nuclear pleomorphism was also noted (Fig. 3). Immunohistochemical staining showed that the tumor cells were positive for the plasma cell markers, Mum-1 and VS38c (Fig. 4), and negative for CD3 and CD20. In addition, expression was was positive for heavy chain immunoglobulin M. The complete blood cell count and serum levels of calcium, creatinine, uric acid and β2 microglobulin were within normal limits. Electrophoresis of serum and urine specimens did not reveal any monoclonal paraprotein and a whole-body bone survey revealed no detectable osteolytic lesions. A bone marrow aspiration was arranged and a plasma cell count of <1% was noted. A few enlarged cervical lymph nodes were also noted bilaterally, and the biopsy of the cervical lymph nodes showed non-specific inflammatory reactions. A final diagnosis of EMP of the nasopharynx was determined, and following tumor excision, the patient underwent radiotherapy with 5,040 cGy in 28 fractions in the nasopharyngeal field. Repeated serum and urine electrophoresis subsequent to 3 months revealed no M protein.

## Discussion

Plasmacytomas have been classified into 3 subtypes. The most common type is multiple myeloma, which is usually a disseminated disease and is characterized by abnormal M protein. The other 2 types, solitary plasmacytoma of the bone and EMP of the soft tissue, are considerably less common. EMPs present in <5% of plasma cell neoplasms and often (>80%) originate in the head and neck region (7). EMPs represent ~4% of nasal cavity tumors and 0.4% of all head and neck malignancies. The diagnosis of EMP of the soft tissue has been based on the following criteria: i) Pathological tissue evidence of monoclonal plasma cells involving a single extramedullary site; ii) no bone marrow involvement; iii) negative skeletal survey results; iv) no anemia, hypercalcemia or renal impairment caused by plasma cell dyscrasia; and v) low serum or urinary levels of monoclonal immunoglobulin (8). The M protein is detected in the serum and/or urine of <25% of patients. There is a greater male preponderance (male:female ratio, 3:1), and EMP tends to occur during the fifth and seventh decades of life, rarely being diagnosed in younger patients. In the head and neck region, the majority of EMPs occur as a solitary tumor and ~10% are multiple. Only four cases of EMP have been previously reported in relatively young patients: i) Two 3.5-year-old males with unexpected EMP following adenoidectomy for chronic rhinosinusitis (9); ii) a 12-year-old female who presented with progressive hoarseness and was subsequently found to have EMP coexisting with localized amyloidosis involving the larynx (10); and iii) an 11-year-old male who presented with an EMP of the orbit (11). In the present patient, the tumor was localized in the nasopharynx. There was no involvement of the bony structure or bone marrow and the diagnosis of solitary EMP of the nasopharynx was confirmed. To the best of our knowledge, this is the youngest case of nasopharyngeal EMP to be reported in the literature.

The etiology of this disease remains unknown, but factors such as viral pathogenesis and chronic irritation from inhaled irritants have been previously indicated (12–14). Radiotherapy remains the mainstay for the management of EMP. Previously, Susnerwala *et al* proposed a pathological grading system based on the multiple myeloma grading criteria; tumors classified into low, intermediate and high grades, which have been found to correlate closely with outcomes. The study recommended the use of adjuvant chemotherapy in patients with higher-grade disease (14). In general, EMPs are considered radiosensitive, with a local control rate of 90–100% (15). A radiation dose of 40–50 Gy delivered to the primary site of the EMP in the nasopharynx is usually recommended (8). Irradiation to the neck is required only in cases with clinically positive cervical node metastasis. In a recent study by Sasaki *et al*, it was found that radiotherapy was quite effective and safe for patients with EMP in the head and neck region. Moreover, radiotherapy combined with surgery produced an improved outcome, as determined by survival rates (12). Although the role of chemotherapy in EMP treatment has not been established, chemotherapy is usually considered for EMPs with high risk factors for local treatment failure (tumor size of >5 cm) and in cases of refractory disease (8). Follow-up radiological and electrophoresis assessment is required following treatment to detect recurrence and progression to multiple myeloma, which occurs in 10–30% of cases. The overall 10-year survival rate is ~70% (7,8).

A literature search revealed no publications supporting the use of surgery alone to treat EMP. In the current case, although the tumor was well defined and thus completely excised, and the patient recovered from the surgery smoothly, subsequent irradiation was recommended. Only four cases of EMP have been previously reported in relatively young patients. To the best of our knowledge, the present case is the youngest case of nasopharyngeal EMP to be reported in the literature.
